# Elementary Atlas of *Drosophila melanogaster* Mutations

**DOI:** 10.1590/1678-4685-GMB-2022-0211

**Published:** 2022-10-17

**Authors:** Paulo A. Otto

**Affiliations:** 1 Universidade de São Paulo, Instituto de Biociências, Departamento de Genética e Biologia Evolutiva, São Paulo, SP, Brazil.

This is a short notice announcing the publication of an e-book by the Brazilian Society of Genetics (SBG) and reviewing it. The publication ("Elementary Atlas of *Drosophila melanogaster* Mutations") is a direct descendent of the computer program "Drosophila Viewer" (Otto, 2000a,b) which is still available at the author's internet site https://www.ib.usp.br/~otto/drosoview.html). The program was designed to run on Windows versions 3.1 and 95 and still works on Windows XP, but on modern PC computers with the latest versions of Windows it will run only when loaded into virtual partitions able to contain older versions of Windows.

 The contents of the e-book, like those of the program "Drosophila Viewer," with a focus on non-specialized audiences, should interest primarily students and professionals specialized in the teaching of Genetics.

 At approximately the same time the program "Drosophila Viewer" was produced, the internet online resources on *Drosophila* genetics, anatomy, development and molecular biology grew substantially, especially those from the FlyBase organization (http://flybase.bio.indiana.edu/), sponsored by the University of Indiana at Bloomington, Indiana. The FlyBase site houses an incredible number of files dedicated to the detailed, in-depth biology of *Drosophila*, including the excellent collection of photographs of classical phenotypic mutants by Holtzman and Kaufman (2013). The (also excellent) “Learning to Fly” poster by Childress *et al.* (2005) can also be accessed in the internet. The photos from this poster inspired many divulgation works of phenotypic mutations in the fly, such as the cornerstone 2013 book “Atlas of Drosophila Morphology,” by Chyb and Gompel, with photographs obtained [just like the images of Holtzman and Kaufman (2013) and Childress *et al.* (2005)] by using state-of-the-art Leitz's Leica multifocal equipment and a careful setup of living flies with phenotypic mutations.

In order to keep alive the graphic material from the "Drosophila Viewer" program and organize everything as an elementary Atlas on phenotypic mutations of *Drosophila*, the e-book was produced by working on all the original drawings used in the program and adding material obtained with permission from other sources, especially from Professors Thomas C. Kaufman (photos by Holtzman & Kaufman 2013 at the FlyBase site) and Georg Halder (from the trio Childress *et al.*, 2005 responsible for the "Learning to Fly" poster). The main part of the Atlas is the presentation of 169 different phenotypic mutant genetic variations of the fly *Drosophila melanogaster*, which were selected taking into account their level of conspicuity (most of them can be easily recognized even by neophytes), as well as their availability in reference *Drosophila* research and teaching centers. Each presentation consists of a box containing illustrations and photos of mutants. Each mutant is explained by a short text taken from the public domain *Drosophila* "red book" by Lindsley and Grell (1972).

Each box contains a diagrammatic but detailed color representation of the corresponding phenotypic mutation and some extra material. The color illustrations, taken from the archives of the program "Drosophila Viewer" had the brown color of the flies reworked to bring it to the true color of the wild-type *Drosophila melanogaster*; a few more original color diagrams were produced to represent some new variants from some photographs not included in the computer program or from the photos by Holtzman and Kaufman (2013) and by Childress *et al.* (2005). In addition to the color drawings, most boxes include a black-&-white drawing by Miss Edith M. Wallace (Professor Thomas Hunt Morgan's illustrator), or, less often, the drawing published by a different *Drosophila* research group, who described the mutation. The boxes contain also original color photographs of whole-mounts of *Drosophila* mutants and/or photos of living specimens obtained from the FlyBase repository of Holtzman and Kaufman (2013) and/or from the "Learning to Fly" poster, as well as some few photos from other sources.

**Figure 1 -:**
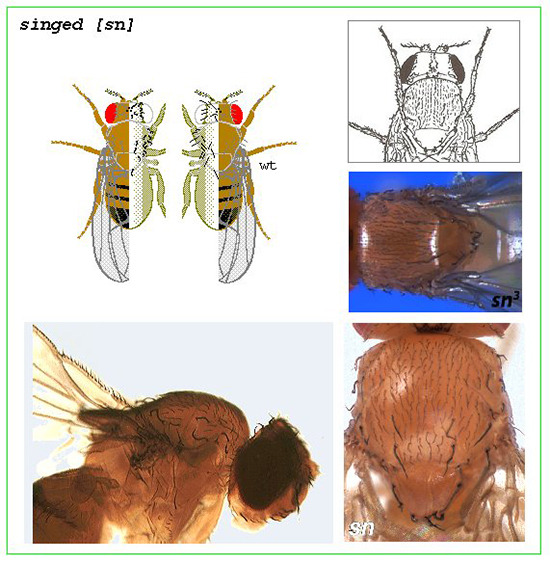
Box corresponding to the mutant phenotypic character *singed* in *Drosophila melanogaster* (details in the text above).

Figure 1 is a sample of such a box. It depicts details on the X-linked (chromosome 1) recessive mutant phenotypic mutant character *singed*. The box contains, upper row, left: an original color illustration taken and repainted from the "Drosophila Viewer" program, showing the bristles in the mutant and in the wild-type fly; upper row, right: a black & white drawing of a mutant fly (detail from the figure in the original publication by Mohr (1922), who described the phenotype in 1922) and a color photo of its dorsal/thoracic aspect (Holtzman and Kaufman, 2013 FlyBase site); lower row, left: original color photograph from a whole-mount slide preparation ("Drosophila Viewer" program); lower row, right: color photo of the thorax of the mutant fly (Childress *et al.*, 2005 “Learning to Fly poster”).

The texts describing the mutants contain only the elements necessary to understand the basic phenotypic expression of each mutant. They were shortened from the complete "red book" text archives in the program "Drosophila Viewer", generally housing additional genetic, cytologic, molecular and developmental details of interest only to specialists. In the case of the mutant phenotype shown in Figure 1, in addition to the information on the illustrations contained in the box, already detailed above and not included below, the corresponding text reads:

*singed [sn]*

Lindsley & Grell, 1972 **, pp. 230-231**

location: 1-21.0.

phenotype: Bristles twisted and shortened. Hairs wavy. Female sterile. Eggs laid are short and have flattened filaments.

**Figure 2 -:**
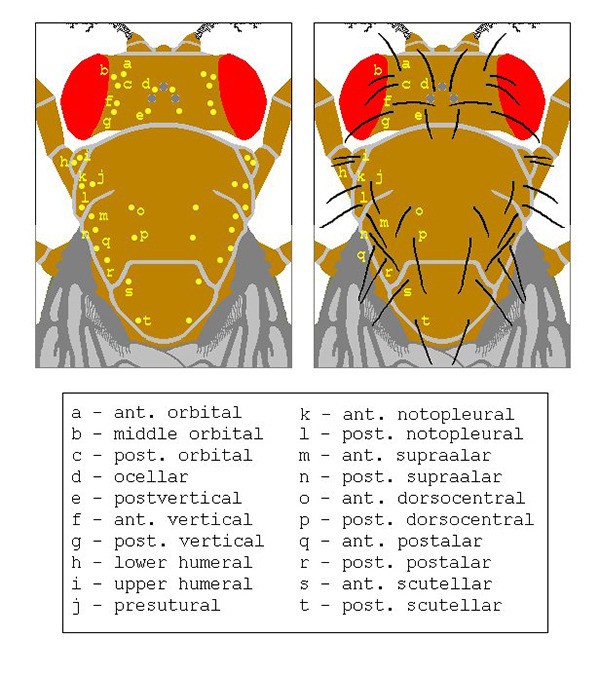
Main head and thorax bristles (details in the text and box above).

**Figure 3 -:**
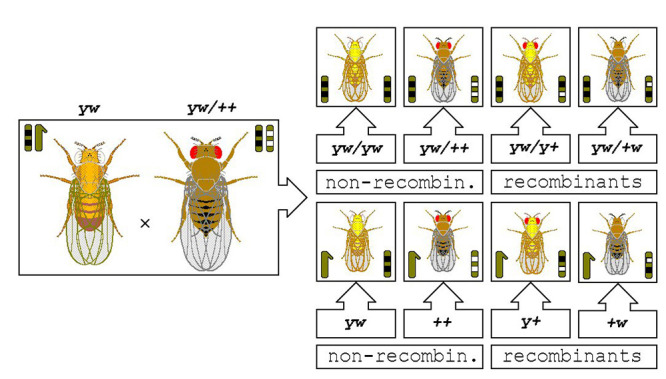
Non-independent assortment of two X-linked mutant recessive characters (details in the text above).

The Atlas also contains some pages with very schematic illustrations of *Drosophila* anatomic and developmental details relevant to the understanding of the phenotypes described in the texts and depicted in the figures and photos, and a series of pictures describing the expected results of typical crossings between some best-known phenotypic mutants of the fly. Practically, all these illustrations were taken or adapted, like the flies' drawings, from the program "Drosophila Viewer". Two examples of such illustrations are shown below (Figures 2 and 3).

Figure 2 shows the insertion, location and orientation of main head and thorax bristles in a wild type fly (upper/dorsal aspect). 

Figure 3 illustrates the non-independent assortment of two X-linked mutant recessive characters, *yellow* and *white*. A F1 wild-type female *yw/++* is crossed to a *yellow white* male *yw*, both out of P wild-type male (*++*) by *yellow* white female (*yw/yw*) crosses. The F2 progeny of such crosses is formed by non-recombinant (females: *yw/yw* and *yw/++*; males: *yw* and *++*) and by recombinant (females: *yw/y+* and *yw/+w*; males: *y+* and *+w*) flies, due to gene recombination that occurred during gametogenesis in their female parent. The different F2 phenotypes depicted occur with expected frequencies that depend on the physical distance between the involved linked genes. 

Formal permission for all extra material included in the Atlas was obtained directly from the respective corresponding authors and editors listed in the Atlas and in the program "Drosophila Viewer", or in the publications describing it (Otto 2000a,b).
